# Using mobile technology to deliver a cognitive behaviour therapy-informed intervention in early psychosis (Actissist): study protocol for a randomised controlled trial

**DOI:** 10.1186/s13063-015-0943-3

**Published:** 2015-09-10

**Authors:** Sandra Bucci, Christine Barrowclough, John Ainsworth, Rohan Morris, Katherine Berry, Matthew Machin, Richard Emsley, Shon Lewis, Dawn Edge, Iain Buchan, Gillian Haddock

**Affiliations:** School of Psychological Sciences, The University of Manchester, Zochonis Building, Brunswick Street, M13 9PL Manchester, UK; Centre for Health Informatics, Institute of Population Health, University of Manchester, M13 9PL Manchester, UK; Health eResearch Centre, Farr Institute for Health Informatics Research, University of Manchester, M13 9PL Manchester, UK; Centre for Biostatistics, Institute of Population Health, The University of Manchester & Manchester Academic Health Science Centre, Jean McFarlane Building, Oxford Road, M13 9PL Manchester, UK; Institute of Brain, Behaviour and Mental Health, Manchester Academic Health Sciences Centre and Manchester Mental Health and Social Care Trust, Manchester, M13 9PL UK

**Keywords:** Psychosis, Randomised controlled trial, Mobile technology, m-health, Connected health, Cognitive behaviour therapy

## Abstract

**Background:**

Cognitive behaviour therapy (CBT) is recommended for the treatment of psychosis; however, only a small proportion of service users have access to this intervention. Smartphone technology using software applications (apps) could increase access to psychological approaches for psychosis. This paper reports the protocol development for a clinical trial of smartphone-based CBT.

**Methods/Design:**

We present a study protocol that describes a single-blind randomised controlled trial comparing a cognitive behaviour therapy-informed software application (Actissist) plus Treatment As Usual (TAU) with a symptom monitoring software application (ClinTouch) plus TAU in early psychosis. The study consists of a 12-week intervention period. We aim to recruit and randomly assign 36 participants registered with early intervention services (EIS) across the North West of England, UK in a 2:1 ratio to each arm of the trial. Our primary objective is to determine whether in people with early psychosis the Actissist app is feasible to deliver and acceptable to use. Secondary aims are to determine whether Actissist impacts on predictors of first episode psychosis (FEP) relapse and enhances user empowerment, functioning and quality of life. Assessments will take place at baseline, 12 weeks (post-treatment) and 22-weeks (10 weeks post-treatment) by assessors blind to treatment condition. The trial will report on the feasibility and acceptability of Actissist and compare outcomes between the randomised arms. The study also incorporates semi-structured interviews about the experience of participating in the Actissist trial that will be qualitatively analysed to inform future developments of the Actissist protocol and app.

**Discussion:**

To our knowledge, this is the first controlled trial to test the feasibility, acceptability, uptake, attrition and potential efficacy of a CBT-informed smartphone app for early psychosis. Mobile applications designed to deliver a psychologically-informed intervention offer new possibilities to extend the reach of traditional mental health service delivery across a range of serious mental health problems and provide choice about available care.

**Trial registration:**

ISRCTN34966555. Date of first registration: 12 June 2014.

## Background

Psychosis is a term used to describe a broad range of experiences such as hallucinations, delusions and confused thinking. Typically, the first episode of psychosis (FEP) occurs between ages 15–35 years [1] and is thought to be a critical period, influencing the long-term course of the disorder. The majority of FEP service users will ‘recover’ within 12-months of treatment. However, the early course of psychosis is characterised by repeated relapse; up to 80 % of service users will relapse within five years of the initial episode. This is significant because each relapse increases the risk of developing persistent-psychotic symptoms and further disconnection from school, work, friends and the community, adversely effecting long-term psychosocial development [2]. A report published by the Schizophrenia Commission [3] found that early intervention for psychosis has the potential to save the National Health Service (NHS) in the UK £125 million over 3 years. Currently, the cost of treating relapsing psychosis is times that of stable psychosis and despite the rise of community care, 70 % of the costs of serious mental health problems are on unplanned inpatient care for relapse. As the early course of psychosis is sharply predictive of the longer-term course of problems, timely, effective and accessible interventions have the potential to prevent the development of sustained and ongoing problems associated with more serious forms of psychosis.

In a recent systematic review and meta-analysis of risk factors for relapse following FEP, medication non-adherence, substance misuse, carers’ critical comments and poor premorbid adjustment (in particular social isolation) were consistently and significantly associated with relapse, defined as exacerbation of positive psychotic symptoms, in FEP. For each of these risk factors, relapse rates increased two-to-four fold [4]. The UK National Institute for Health and Care Excellence (NICE) [5] recommends early intervention services (EIS) for all people with FEP. These services aim to provide a range of treatment options, including pharmacological and psychological interventions. The main treatment for psychosis is medication, which reduces relapse by 75 %. However, more than half of FEP service users do not adhere to medication, and side effects are common [4]. The limitations of pharmacological treatments highlight the importance of evaluating psychosocial interventions in order to improve relapse rates in FEP; the critical period when vulnerability is at its peak [5].

Cognitive behavioural interventions have been the most robustly evaluated psychological approach for psychosis. Wykes and colleagues [6] carried out a meta-analytic review of 34 cognitive behaviour therapy (CBT) trials targeting people with a schizophrenia-related diagnosis across various countries. There were overall beneficial effects for the target symptom in 33 studies (effect size = 0.400; 95 % CI = 0.25, 0.55) as well as significant effects for positive symptoms (32 studies), negative symptoms (23 studies), functioning (15 studies) and social anxiety (2 studies) with effects ranging from 0.35 to 0.44. Overall, results from this meta-analysis indicated a ‘modest’ effect size in improving positive symptoms compared to standard psychiatric care. Jauhar and colleagues [7] recently updated the Wykes et al. [6] systematic review and meta-analysis of CBT for core schizophrenia symptoms and found results from randomised controlled trials (RCTs) were broadly consistent with previous results. Fifty-two studies from various countries were included in the meta-analysis. There was an overall significant but modest impact of CBT on psychotic symptoms, with blinded studies showing lower effect sizes on overall symptoms and positive symptoms, but not for negative symptoms. However, this latter meta-analysis has been criticised for its over-simplification of the complexities of psychosis presentations and psychological interventions [8]. Given the available evidence, CBT is recommended as a first-line intervention by NICE [5]. Despite this recommendation, a shortage of trained clinicians and pressure on resources mean that substantial numbers of people who could benefit do not have access to CBT. In a recent study in the North West of England, < 10 % of people eligible were offered or received CBT [9]. Even those who are offered CBT often experience lengthy delays before receiving treatment, resulting in relapse indicators being missed [3]. Accordingly, there is an urgent need to expand access to helpful psychosocial interventions for psychosis.

Given advancements in mobile phone technology, it is possible to provide ecologically-valid interventions via smartphones. Smartphones have become everyday devices that an increasing number of people routinely keep about themselves. Recent surveys [10, 11] found that the majority of service users with psychosis own and use a smartphone, and the rates of ownership and use are comparable to those of the general population. Given the barriers associated with accessing psychological interventions such as CBT, smartphones offer an unprecedented opportunity to enhance health status by delivering real-time interventions that have the potential to extend the reach of psychosocial interventions. Researchers and technology developers are starting to develop smartphone software applications (apps) for both symptom monitoring and treatment delivery, e.g. [10–13], each of which have shown promising results in terms of feasibility, acceptability, and improvements in their primary outcomes. These studies have not, however, examined preliminary efficacy of the technology using either a controlled trial design or an active control condition.

The aim of our Medical Research Council (MRC) funded Actissist trial is to overcome barriers to the implementation of CBT by developing and implementing a novel, theory-driven, user-informed CBT smartphone app targeting FEP relapse indicators. The current study represents the third phase in the development and testing of the Actissist app. In phase 1, we completed individual interviews with 21 EIS service users and focus groups with 48 EIS clinicians in the North West of England to inform the content of the Actissist app and the protocol design of the trial. These personal accounts were analysed using an approach informed by Framework Analysis. This approach ensured that both the intervention and trial design were user-informed and user-led. Qualitative interviews with services users and staff explored issues including: does Actissist make sense in service users’/staff’s daily life; incentives/barriers to routine use; equity and ethics; privacy/surveillance concerns; acceptable number/frequency of app interactions; and interface option preferences. In the second phase, the Actissist technical team, consisting of software engineers at The University of Manchester Centre for Health Informatics, built the Actissist app over an 8-month period alongside the qualitative period of work. At this third phase, in a RCT, we will test whether Actissist is feasible and acceptable to EIS service users over and above symptom monitoring and usual care. Actissist facilitates users’ self-management of symptoms and intervention delivery, which we hope will empower service users to make informed intervention choices by way of providing opportunities to modify behaviour directly via a ubiquitous interface (smartphone). This approach holds other advantages over routine therapy: it reduces recall bias and overgeneralisation of problems that often occurs during face-to-face therapy, and it allows the *context* of symptom and behaviour change to be assessed.

### Objectives

The primary objective is to determine whether in early psychosis service users a CBT-informed app (Actissist) is feasible to deliver and acceptable to use. Importantly, we will estimate parameters for the design of a future RCT, such as testing recruitment strategies, identifying a suitable primary outcome measure for use in future research, drop-out rates, proportion of eligible participants consenting, proportion continuing for 12 weeks (both arms), proportion of data-points completed across all participants, examining the characteristics of outcome measures and estimating the standard deviation and intra-cluster correlation to aid in future sample size calculation, and collecting information on follow-up response rates. Gathering such information will allow for the estimation of potential treatment effect sizes to inform a larger definitive trial as compared with service users receiving a symptom monitoring app (ClinTouch; [10]). We also aim to evaluate whether the Actissist app: (i) has the potential to impact on predictors of FEP relapse; and (ii) potentially enhances user empowerment, functioning and quality of life. In a future definitive trial, we hypothesise that Actissist will reduce the severity and distress of psychotic symptoms, perceived criticism and cannabis misuse, improve socialisation and quality of life and functioning and facilitate user-empowerment compared to the control condition at each of the follow-up time points. Using qualitative methods we will employ purposive sampling methodology to examine acceptability of the Actissist app.

## Methods/Design

**Fig. 1:**
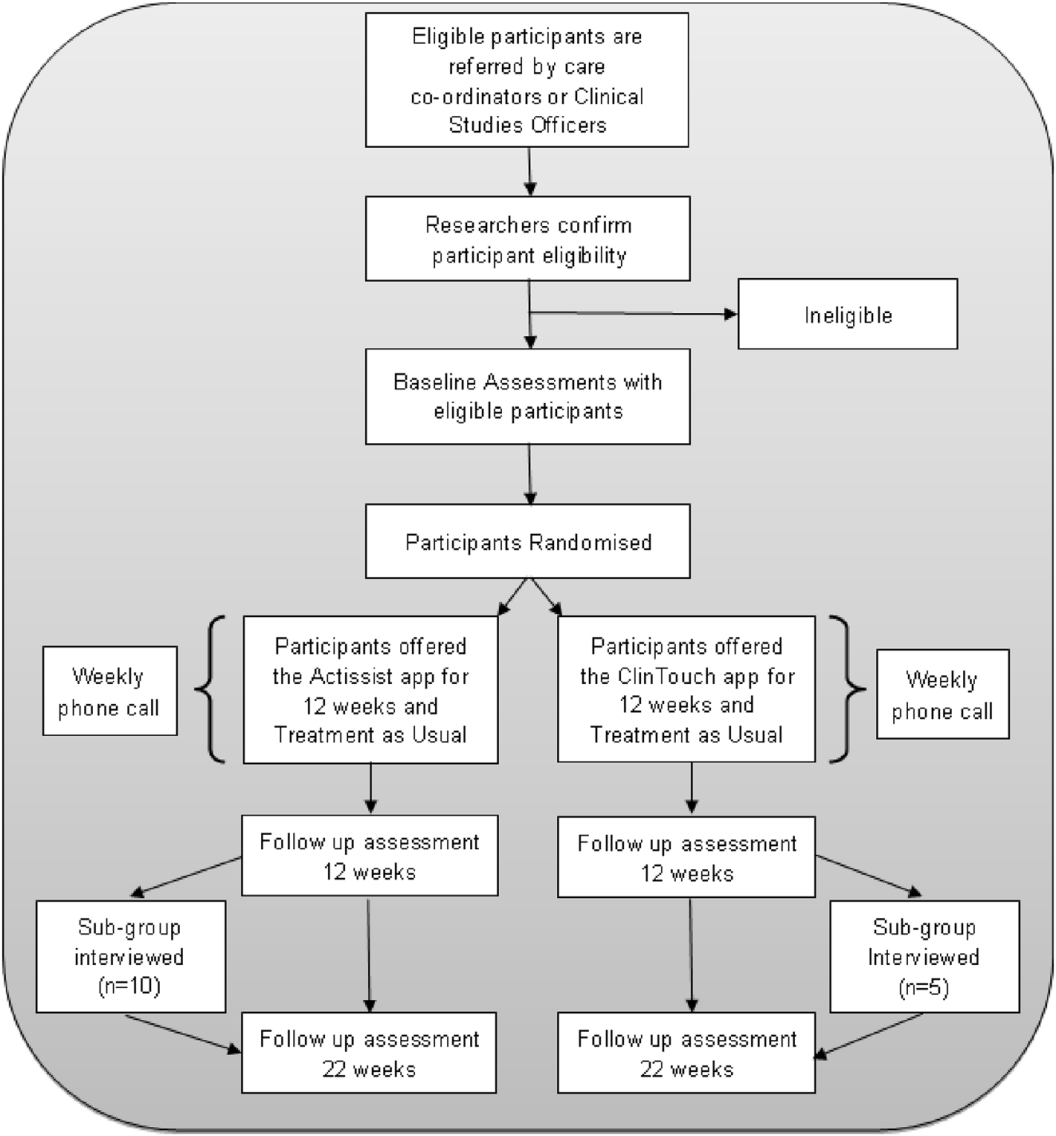
Consolidated Statement of Reporting Trials (CONSORT) diagram showing design of study

The trial is funded by the MRC and has received ethical approval from the National Research Ethics Committee West Midlands – South Birmingham (14/WM/0118). This is a user-informed, single-blind RCT with random allocation to one of two conditions: Actissist + TAU versus ClinTouch + TAU. We will recruit participants registered with early psychosis services in the North West of England, UK. Figure 1 provides a summary of the study procedure. Potential participants are given at least 24 hours to consider the study information before being contacted by a researcher to discuss the study in more detail. For willing participants who meet the inclusion criteria, full written consent will be obtained prior to the baseline assessment.

Following baseline assessment, participants are randomised and informed of their group allocation. Receipt of either app will usually commence within 2 working weeks of randomisation. Blind follow-up assessments will occur at 12 weeks after baseline (post-treatment) and 22 weeks after baseline (10 weeks post-treatment). We will use both qualitative and quantitative methods to evaluate whether the aims of our study are met, ensuring that participant and staff views are adequately captured. Over a 6-month recruitment period, a total of 36 consenting EIS service users will be randomly allocated at a ratio of 2:1 to Actissist + TAU (*N* = 24) or ClinTouch + TAU (*N* = 12). Since this is a feasibility study, we aim to find out as much information as possible from service users who are allocated to and use the Actissist intervention. Therefore, we ensure that most of our service users in the study are allocated to Actissist by using a 2:1 allocation ratio.

### Measures

For the primary outcome, we will assess feasibility in terms of uptake (the proportion of eligible participants consenting to join the study), attrition rate, the proportion of participants completing user and alert-initiated data entries across participants (>33 % data points) and participant feedback. Secondary outcomes include psychotic and mood symptoms, perceived criticism, cannabis use, medication adherence and socialisation, as well as user-empowerment, quality of life and functioning. Trained raters will be supervised throughout the study to ensure reliability and validity of the interview administered assessments. Outcomes will be assessed by raters blind to treatment allocation.

#### Psychotic symptoms

The Positive and Negative Syndrome Scale (PANSS; [14]) includes scales of positive symptoms, negative symptoms and general psychopathology and is used widely in psychosis research. The Psychotic Symptoms Rating Scales (PSYRATS; [15]) is a well-validated assessment of the frequency and intensity of hallucinations and delusions in psychosis and associated distress. The scale has excellent psychometric properties with inter-rater reliability for the scales ranging between 0.78–1.0 [16]. Raters will be trained to a ‘gold standard’ and reliability will be assessed for the duration of the trial.

#### Depression

The Calgary Depression Scale for Schizophrenia (CDSS; [17]) is a nine-item observer rated measure specifically designed for schizophrenia, minimising contamination by negative symptoms and the extrapyramidal side effects of neuroleptics. It is strongly correlated with the Beck Depression Inventory (BDI; [18]) (*r* = 0.91) and is responsive to change in psychosis [19].

#### Functioning

The Global Assessment of Functioning scale (GAF: [20]) is a standardised measure used to assess overall level of functioning. The Personal and Social Performance Scale (PSP; [21]) is a single-item rating scale based on assessments of function in four main areas: socially useful activities, personal and social relationships, self-care, and disturbing and aggressive behaviours. The PSP has excellent reported inter-rater reliability (intra-class correlation coefficient = 0.98; 21).

#### Empowerment

The Empowerment Rating Scale (ERS; [22]) is a 28-item scale designed to measure empowerment in users of mental health services. The scale has good internal reliability (Cronbach’s alpha = 0.86).

#### Health status

The EuroQol-5D-5 L (EQ-5D-5 L) [23] is a measure of health status and health-related quality of life. Participants are requested to: i) rate their own health state on 5 dimensions (mobility, self-care, usual activities, pain/discomfort and anxiety/depression); and ii) rate their current health status on a thermometer ranging 0–100.

#### Substance misuse

Frequency and quantity of alcohol and cannabis misuse is assessed using the timeline follow back scale (TLFB; [24]), which has good reported reliability and validity in clinical and non-clinical populations, including psychosis [25]. The TLFB procedure reconstructs daily substance use over a specified time period (90 days) by detailed inquiry from an interviewer and use of a calendar with salient prompts to aid recall.

#### Criticism

The Perceived Criticism scale (PC) [26] is a measure of perceived criticism by a significant other. Participants are asked to rate how critical they think their significant other is of them using a 10-point Likert scale. Previous research suggests that the PC scale has good predicative validity and is not correlated with current symptoms of depression [27] or anxiety [28].

#### Medication adherence

Medication adherence and attitudes to medication will be measured by the Medication Adherence Rating Scale (MARS; [29]), which is a well-validated self-report questionnaire including items regarding adherence behaviour and attitudes to medication.

#### Satisfaction with technology

A Quantitative Feedback Questionnaire (QFQ; [30]) will be given to service users at the post-treatment follow-up assessment. The QFQ was developed to assess the acceptability and feasibility of utilising a smartphone application (ClinTouch) within a service user sample. The QFQ consists of 27 items pertaining to the integration of the technology into the daily routine and methodological reactivity.

### The intervention – Actissist

The Actissist intervention is grounded in the cognitive maintenance model of psychosis, which proposes that cognitive appraisals contribute to the emergence of unhelpful beliefs and influence the interpretation of psychotic experiences [31, 32]. Distress is largely linked to the meaning and interpretation of symptoms and beliefs regarding anticipated consequences. In developing the intervention, we incorporated key theoretical elements from Morrison and Barratt’s [33] Delphi Study on the core components of CBT for psychosis and Roth and Pilling’s [34] competence framework for psychological interventions for psychosis. Specific functions of the app were informed by CBT content described in various published academic works, e.g. [35–44]. We also drew on experience sampling methods and the clinical protocols described by Granholm and colleagues [13] and Ben-Zeev and colleagues [11]. Importantly, we gathered views and insights from research with service users and EIS clinicians, expert clinical academics and software engineers during phase 1 of the overall project to more specifically inform the intervention.

The Actissist app targets five domains: perceived criticism; socialization; cannabis use; paranoia; and distressing voice-hearing. Medication adherence is monitored on a weekly basis. The Actissist intervention protocol works as follows: the app emits an alarm prompting participants to access the app at 3 pseudo-randomised time points per day, 6 days a week between the hours 10 am to 10 pm for 12-weeks alongside usual treatment. The prompts serve as a reminder to use the app and requests that the participant accesses one/more of the target treatment domains (if applicable). Participants can either snooze (for up to 30 minutes) or decline interacting with the app at any given alarm alert. Participants can also self-initiate use, providing them with the flexibility to engage with Actissist as and when required. Participants complete a series of self-assessment questions that are structured as question-answer exchanges, which are then followed by normalising messages and cognitive and behavioural strategies for managing distressing experiences. The participant’s response to the cognitive appraisal selected within each treatment domain question-answer exchange determines the normalising messages and cognitive and behavioural suggestions that follow. There are multiple messages associated with each branched response to minimise boredom and repetition within the app. In addition to the self-assessment questions, participants can access a repository of multi-media material, including audio relaxation and mindfulness exercises, audio-visual service user recovery stories, an open diary, an array of mental health fact sheets, external links to web-related content (e.g. Technology, Entertainment and Design (TED) talks, useful websites), and a graphical summary of data points entered (previous 7 days) for each domain accessed. Users can customise the aesthetics of the Actissist interface; personally meaningful images from the smartphone’s local storage can be set as the app’s wallpaper to facilitate positive memory recall and/or positive mood induction.

Prototypes of the app were produced in bounded development iterations and reviewed by the research team, members of an expert reference group, clinicians and service users who also provided qualitative feedback on the user interface and software performance/usability, with feedback incorporated into the next iteration of the system development. The software was beta-tested with service users, clinicians and academics over a 7-day period. Beta testers provided qualitative feedback regarding multiple aspects of the system and the user interface.

### Control condition – ClinTouch

The ClinTouch app (control condition) is a symptom monitoring app that triggers, collects and wirelessly uploads symptom data to a server. As in the treatment condition, the app emits an alarm prompting participants to access the app at 3 pseudo-randomised time points per day, 6 days a week between the hours 10 am to 10 pm for 12 weeks alongside usual treatment. The ClinTouch protocol is outlined in detail in Palmier-Claus et al. [10]; although, the number of beeps is altered here for parity with Actissist alerts, such that participants submit 1.5 data points daily with 10 branching items covering positive psychotic symptoms, anxiety and mood. As each full data point is collected over two separate alerts, this equates to receiving three alerts every day. Participants are asked to indicate the degree to which they agree/disagree with 10–18 symptom-statements since their last entry. A one-off ‘snooze’ option is available if participants are occupied.

### Treatment As Usual (TAU)

TAU for EIS service users typically involves regular meetings with a care co-ordinator, access to a psychiatrist, medication and monitoring of risk that requires immediate action and psychosocial interventions (including CBT) as required/desired. Treatments, including psychosocial interventions, are not withheld in the current study for ethical reasons. Instead, we monitor other interventions received during the trial intervention period during a weekly phone call with participants.

For both conditions, participants are trained by the trial research assistant (RA) in how to use the app and participants view written ‘in-app’ instructions and receive a printed hard copy of instructions about how to use the app. Developed for the Android operating system, software is pre-loaded on a loaned smartphone or downloaded on the participant’s own smartphone. The project officer will phone all participants weekly to remind them to charge the device and troubleshoot any other practical issues that might arise. Concerns will be managed in close consultation with participants’ care co-ordinators and emergency contacts will be clearly available in the menu options on both apps. Following completion of baseline assessments, participants are randomised within 2 working days.

### Inclusion and exclusion criteria

Participants meeting the following criteria will be eligible for the study: i) in current contact with an EIS in the North West, UK; ii) capacity to provide informed consent; iii) sufficient English language proficiency to complete questionnaires and respond to written material. Exclusion criteria include: i) anyone less than 16 years old at the point of recruitment; ii) not capable of giving informed consent; iii) non-English proficient; iv) anyone currently an inpatient at the time of recruitment. We have left inclusion criteria as broad as possible in order to assess whether different factors influence the feasibility and acceptability of the Actissist intervention, and improve the external validity of the trial.

### Recruitment and randomisation

Participant recruitment is taking place across a number of NHS Trusts across the North West, UK. Advertisements, including flyers and posters to promote the study are distributed throughout a range of NHS services. The trial also has a website to advertise the study (www.psych-sci.manchester.ac.uk/actissist/). In addition, researchers present verbal and written information outlining the study to health professionals. Potential participants are offered a participant information sheet along with an overview of the study by either a member of their care team or directly by the researcher. Eligible participants are identified by care co-ordinators and approached by a trained RA or clinical studies officers (CSO) from the UK Clinical Research Network (CRN). Participants deemed eligible to participate by the care co-ordinators are invited to take part and asked to provide informed consent. Once informed consent has been obtained, trained researchers administer a battery of assessments and upon completion of the assessments participants are randomly allocated in a 2:1 ratio to the Actissist + TAU group or the ClinTouch + TAU group. The study statistician produced a randomisation list using random permuted blocks of sizes 3 and 6. Notification of group allocation is conducted using an independent tool (eLabs; nweh.org.uk/content/elab), an online research technology platform developed and managed by The University of Manchester in order to ensure concealment of group allocation. Group allocation is revealed to the participant, clinician, baseline research assessor and project officer. Blindness of raters is ensured using a variety of procedures, including briefing participants prior to assessment not to disclose their allocation and data protection of randomisation information. The baseline RA is unblinded as they are required to conduct a training set-up session with participants on how to use the allocated app post-randomisation. Unblindings will be regularly monitored and recorded. Deliberate unblinding would only occur in the case of a serious adverse incident such as risk towards self/others by a participant.

### Data monitoring and management

Study data will be entered onto study specific SPSS (SPSS Inc., Chicago, IL, USA) database following a standard operating procedure. A separate database will be used for each time point, and this will be locked once all participants have been entered. All data will be collected on paper case report forms (CRF) that are anonymised and stored at The University of Manchester in locked filing cabinets separate from identifying information. Data will be entered by researchers electronically into the SPSS database. A random subset of data will be checked for quality control by independently checking the paper CRFs and electronically entered data and any errors will be corrected. Only the lead investigators, trial statistician and researchers will have access to the final dataset. If any participant withdraws from intervention, we will seek their permission to collect outcome data. We allow a 2-week period for gathering follow-up assessment data, after which they are coded as missed and re-contacted for the next follow-up assessment point.

Serious adverse events are regularly monitored and documented by the research team and reported immediately to the chief investigator and/or a senior clinical member of the team. Any identified adverse event is then discussed with a nominated senior clinical academic independent to the University of Manchester and the research team and an appropriate course of action is agreed and implemented.

### Participant reimbursement

Some participants in the Actissist and ClinTouch conditions will choose to borrow a smartphone from the researchers, which will be pre-loaded with £10 and then topped up £10 remotely each month to support data connectivity to last the duration of the study. To compensate for their time and contribution, all participants will be reimbursed £20 for completing each assessment time point. In order to ensure that we are covering the data usage costs associated with a participant using their own handset, we will remunerate participants with £10 every month (total of £30), if such costs are incurred. A £10 shopping voucher per fortnight will be given to participants over the intervention period who complete at least onethird of data entry points.

### Privacy and confidentiality

Maintaining service user and clinician trust when using technology is paramount and requires careful consideration when developing technology-based studies. We do not store any identifying data on either the app or the server and, therefore, many security risks are minimised. Any study data stored on the phone by the participant will be accessible only through the Actissist app. We also recommend that service users set a passcode to access their smartphone. Uploading data to a central server in real-time enables study data to be captured and so protects against data loss should a phone be lost or stolen and removes the need for personal data storage on the device. Furthermore, three general principles of information security (confidentiality, integrity and availability) will be followed in the design and implementation of the trial. All data transmitted to and from the servers will be encrypted over https with strong ciphers as detailed in the Approved Cryptographic Algorithms Good Practice Guidelines (REDCap; [45]). Cipher Suites will be implemented in compliance with Section 6 (‘Preferred uses of cryptographic algorithms in security protocols’) of the Good Practice Guidelines. In cases where participant data is downloaded from the Actissist or ClinTouch sites, this data will be securely encrypted with a pass phrase of appropriate length and complexity. Data transfers are secured by using standard web security protocols (TLS).

### Sample size

Since hypothesis testing is not the objective of this study, formal power calculations are not appropriate. In order to estimate the standard deviation of our outcome variables to inform a future sample size calculation, sample sizes between 24 and 50 have been recommended [46–48]. Our proposed sample size of 36 is within the range recommended in the literature and is sufficient for establishing feasibility and obtaining parameters to inform a robust power calculation for such a later trial.

### Statistical analysis

Our primary analysis for a future trial would be by an intention-to-treat (ITT) approach using all randomised participants and report data in line with the Consolidated Standards of Reporting Trials (CONSORT) 2010 Statement [49] showing referral and attrition rates (i.e. participant flow). The primary analysis will involve tabulated and associated graphical summaries of the feasibility outcomes in each randomised group. To inform potential effect sizes for a future definitive trial we will use linear regression to examine the effect of random allocation on the secondary outcomes at post-treatment, adjusting for outcome measures at baseline. Presentation of the analysis will focus on point estimates and associated 95 % confidence-intervals rather than statistical significance (*P* values). Every effort will be made to follow-up all participants in both arms for assessments, and the analysis will use, where appropriate, statistical techniques for handling missing data. Although our focus will be on piloting the use of the study outcome measures, demographic information and quantitative elements of the semi-structured questionnaire will be analysed using descriptive statistics.

### Semi-structured interviews and qualitative analysis

At the end of the trial, a qualitative study will be undertaken to assess the acceptability of Actissist. A purposive sample will be drawn from study participants to ensure maximum variation of sample and to elicit a broad range of views regarding the acceptability of the Actissist app. This will include a balance of participants who have had a good outcome, those who have dropped out of the intervention or who refused participation, and control participants. Interviews will explore participants’ experiences and expectations of the app, participants’ views on the content, duration and intensity of the app (particularly focusing on perceptions of usefulness, engagement with the technology and facilitators/barriers to implementation), the role of an app like Actissist within the management of mental health problems, perceived benefits of the app, disappointments/concerns and ongoing support needs. All interviews will be digitally-recorded, transcribed, checked for accuracy and analysed using thematic content analysis within a qualitative methodological framework [50]. Interviews will be carried out until data saturation is complete. We will ask participants, as well as our expert reference group, to review and verify themes, usually referred to as ‘member checking’ or ‘participant verification’. Additionally, the clinical members of our research team will rigorously review the research process. This peer verification process (together with member checking) is a recognised method of ensuring trustworthiness of the data and subsequent findings [51]. Records of field notes will be maintained and reflections providing adjunctive data will be used to illuminate and justify interpretative decisions. NVivo [52] qualitative software package (QSR International Ltd., Daresbury, UK) will be used to support data management and analysis.

## Discussion

The protocol detailed above is designed to test the feasibility, acceptability and proof of concept of a smartphone app intervention for early psychosis. Digital technology has the potential to transform mental health care. To the best of our knowledge, there are no RCTs of smartphone apps delivering a psychological intervention for early psychosis. For young people in particular (‘digital natives’), it is important to develop technology-based approaches as a way of connecting with individuals about mental health issues; young people can be reluctant to seek professional help for various reasons, including stigma, embarrassment and poor recognition of symptoms. The lack of access to psychological therapies for psychosis stresses the importance of considering innovative methods and solutions to deliver support to people in need in a timely manner. We envisage Actissist as a reusable platform capable of delivering interventions for many problems. As such, we see Actissist as serving not only a clinical need but also a product development need for a platform technology to deliver self-management for a wide range of psychological disorders.

Mobile, wearable and ubiquitous technologies are advancing at an unprecedented rate. Therefore, a major research challenge is being able to rigorously evaluate mobile health (mHealth) interventions using robust scientific methods, such as RCTs, appropriately. One possible way to evaluate mHealth interventions is to adopt more sophisticated trial methodology. In standard RCTs, the intervention is fixed at the onset of the trial and is not permitted to evolve during the trial duration. Indeed, for many drugs under investigation or complex interventions, this is reasonable. However, for digital interventions, this is problematic due to the pace of change in such interventions; fixing the intervention at trial onset can render it obsolete by the time the trial results are available. Apps can also be costly and can time consuming to develop, such that the app might be out-dated by the time it is completed. Therefore, ideally, trials of digital interventions need to be adapted to allow the intervention, and potentially the control arm, to evolve as the trial progresses. Another anticipated challenge in the mHealth field is that low-income individuals may not be able to afford smartphones or indeed the sufficient levels of data necessary to run apps and other smartphone functions. To overcome this potential problem, we provide participants with mobile phone handsets and cover data network charges. However, factors such as these could be a practical barrier to continuous mHealth services. From a psychological perspective, therapeutic alliance is a key predictor of outcomes in psychological therapy [53]. Mobile technology has been criticised for lacking this essential therapeutic ingredient. Nonetheless, there is an emerging literature regarding the concept of therapeutic alliance in the context of electronic health (eHealth) and mHealth ranging from alliance service users may form with any therapist supporting the technology to ‘relationships’ that service users may form with mobile devices or apps themselves [54]. We propose to contribute to this emerging literature by developing a measure of alliance to the Actissist app and determining the feasibility of administering the new measure. Furthermore, if trials such as Actissist are effective, a major challenge is for mental health services to recognise and incorporate digital interventions into mainstream health service delivery. Indeed, compatibility issues could pose significant barriers to real-world implementation due to the fast paced development in mobile technology and platforms. One possibility for future research following this trial would be to run a pragmatic trial of the Actissist intervention in routine mental health services. Importantly, the current study has had strong user and clinician involvement from the ground up: we see service users as essential co-designers of mobile mental health interventions and essential co-investigators in trials. In sum, we believe Actissist is an important example of experimental medicine; it is a novel intervention developed from an empirically-derived theoretical framework. This study represents an important and significant step towards developing a technology platform for delivering a range of psychosocial interventions for serious mental health problems.

## Trial status

Recruitment commenced end March 2015 and is expected to end September 2015. The first participant was randomised April 2015.

## Abbreviations

Actissist: Active Assistance for Psychological Therapy
app: mobile phone software application
BDI: Beck Depression Inventory
CBT: cognitive behaviour therapy
CDSS: Calgary Depression Scale for Schizophrenia
CI: confidence interval
CONSORT: Consolidated Standards of Reporting Trials
CRF: case report forms
CRN: Clinical Research Network
CSO: clinical studies officer
eHealth: electronic health
EIS: early intervention service
EQ-5D-5 L: EuroQol-5D-5 L measure of quality of life
ERS: Empowerment Rating Scale
FEP: first episode psychosis
GAF: Global Assessment of Functioning scale
ITT: intention-to-treat analysis
MARS: Medication Adherence Rating Scale
m-Health: mobile health
MRC: Medical Research Council
NHS: National Health Service
NICE: National Institute for Health and Care Excellence
PANSS: Positive and Negative Syndrome Scale
PC: perceived criticism
PSP: Personal and Social Performance scale
PSYRATS: The Psychotic Symptoms Rating Scales
QFQ: Quantitative Feedback Questionnaire
TLFB: timeline followback scale
RA: research assistant
RCT: randomised controlled trial
TAU: Treatment As Usual
TED: Technology, Entertainment and Design
UK: United Kingdom
